# Role of GRK2 in Trophoblast Necroptosis and Spiral Artery Remodeling: Implications for Preeclampsia Pathogenesis

**DOI:** 10.3389/fcell.2021.694261

**Published:** 2021-11-30

**Authors:** Zi Lv, Li-ling Xiong, Xian Qin, Hua Zhang, Xin Luo, Wei Peng, Mark D. Kilby, Richard Saffery, Philip N. Baker, Hong-bo Qi

**Affiliations:** ^1^Department of Obstetrics, The First Affiliated Hospital of Chongqing Medical University, Chongqing, China; ^2^State Key Laboratory of Maternal and Fetal Medicine of Chongqing Municipality, Chongqing Medical University, Chongqing, China; ^3^Joint International Research Laboratory of Reproduction and Development of Chinese Ministry of Education, Chongqing Medical University, Chongqing, China; ^4^Department of Obstetrics and Gynecology, The First Affiliated Hospital of Chongqing Medical University, Chongqing, China; ^5^Centre for Women’s and New Born Health, Institute of Metabolism and Systems Research, University of Birmingham, Birmingham, United Kingdom; ^6^Cancer, Disease and Developmental Epigenetics, Murdoch Children’s Research Institute, Parkville, VIC, Australia; ^7^College of Medicine, Biological Sciences and Psychology, University of Leicester, Leicester, United Kingdom

**Keywords:** GRK2, necroptosis, preeclampsia, trophoblast, cell death

## Abstract

Impaired invasion of extravillous trophoblasts and severe oxidative stress manifest the poor placentation in preeclampsia, which is life-threatening and more than a hypertensive disease of pregnancy. Previous studies have reported that G protein-coupled receptor kinases (GRKs) play a key role in initiating hypertension and hypertensive renal damage, yet little evidence so far suggests a link between GRKs and preeclampsia-related hypertension. Here, we demonstrate GRK2 expression is significantly downregulated (*P* < 0.0001) in preeclamptic placentae compared to normotensive controls. Knockdown or inhibition of GRK2 in placentae caused insufficient arterial remodeling and elevated trophoblast necroptosis *in vivo*. These further induced preeclampsia-like phenotype in mice: hypertension, proteinuria, and elevated pro-angiogenic cytokines. By human extra-villous invasive trophoblast cell line (HTR8/SVneo cells), we revealed the knockdown or inhibition of GRK2 triggered excessive death with typical necroptotic characteristics: nuclear envelope rupture and the activation of RIPK1, RIPK3, and MLKL. Necrostatin-1, an inhibitor of RIPK1, is able to restore the survival of trophoblasts. Together, our findings demonstrated that insufficient GRK2 activity compromises spiral artery remodeling and initiates necrotic events in placentae, thereby leading to preeclampsia. These findings advance our understanding of GRK2 in the pathogenesis of preeclampsia and could shed light on a potential treatment for preeclampsia.

## Graphical Abstract

Lv et al. show that trophoblastic GRK2 deficiency could promote placenta dysfunction and PE-like phenotype by activating necroptosis in trophoblasts, then inducing cytokine disturbance in circulation.

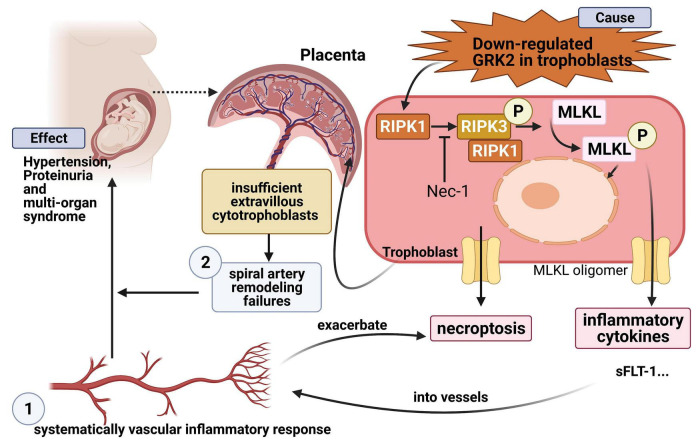

## Introduction

Maintaining normal blood pressure is often a significant challenge for pregnant women. Preeclampsia (PE) is a life-threatening disease characterized by new-onset hypertension (>140/90 mmHg), proteinuria (>300 mg/L/day), and/or end-organ complications after 20 weeks of gestation. PE results in an estimated 76,000 maternal deaths and 500,000 fetal and newborn deaths annually (von Dadelszen et al., 2020). The precise cause of PE remains elusive, but the placenta is generally agreed to play a key role, as the clinical symptoms of PE are rapidly relieved after delivery and the removal of the placenta (Apicella et al., 2019). The typical model of PE is presented in two stages: the placental and systemic effects (Redman, 1991, 2005; Staff, 2019). The initial stage arises from poor placentation. Fetal cytotrophoblasts differentiate into endothelial extravillous cytotrophoblasts (EVTs), which invade and dilate uterine spiral arteries (Paria, 2002). Poor placentation with abnormally small spiral arteries results in insufficient access to nutrients in the maternal blood (Labarrere et al., 2017). Consequently, localized ischemia and oxidative stress occur (Spradley et al., 2016; Torres-Cuevas et al., 2017; Tong and Giussani, 2019), which further promotes excessive trophoblast cell death and the release of bioactive products into the maternal circulation (Makris et al., 2007; Uchida, 2013). In the second stage of PE, the cardiovascular and renal features of the condition are suggested to be an effect of placental ischemia and spiral artery insufficiency (Melland-Smith et al., 2015; Harmon et al., 2016; Wu et al., 2017).

Exaggerated trophoblast cell death may play a significant role in poor placentation. AnnexinA7 (ANXA7) was reported to be involved in placental apoptosis through the ANXA7/JAK1/STAT3 pathway, and it may also contribute to the generation of oxidative stress in PE placentae (Mo et al., 2019). This notion has been corroborated by findings of increased ADMA levels and enhanced sensitivity to apoptotic cytokine TRAIL in PE placentae (Lumicisi et al., 2015). Notably, the apoptotic mechanism has been well conserved in a “silent and passive” manner that commonly avoids organ injuries, while PE placentae are characterized by injuries like intervillous thromboses and infarctions (Yougbaré et al., 2017; Patel, 2018). Necroptosis, a novel form of cell death, is associated with the release of inflammatory cytokines and may be more likely to affect poor placentation (Kim et al., 2019; Nailwal and Chan, 2019). In necroptosis, receptor-interacting serine-threonine kinase 1 (RIPK1) and receptor-interacting serine-threonine kinase 3 (RIPK3) interact, before activating mixed-lineage kinase domain-like protein (MLKL), the executor that causes cell lysis (Khan et al., 2021). However, research regarding necroptosis-associated effects on poor placentation of PE remains limited.

G protein-coupled receptor kinases (GRKs) play a key role in regulating blood pressure (Belmonte and Blaxall, 2011). GRK3 expression in human lymphocytes is inversely correlated with blood pressure (Oliver et al., 2010), whilst GRK4 gene variants induce sodium retention *via* renal dopamine receptor impairment (Chen et al., 2014). Also, GRK2 has been proven to act as a “gatekeeper” for cardiovascular diseases in a GPCR-independent manner (Izzo et al., 2008), and GRK2 knockdown in mice results in age-dependent spontaneous hypertension (Tutunea-Fatan et al., 2015). GRK2 was also reported to have a protective role in apoptosis and the production of mitochondria-related reactive oxygen species (ROS) (Fusco et al., 2012), whereby the acute accumulation of GRK2 in mitochondria prevented ischemia-reperfusion injury both *in vitro* and *in vivo* (Penela et al., 2010; Theccanat et al., 2016; Sato et al., 2018). Moreover, the Elabela/Apelin-APJ system, which have been proven to role in mid/late gestation angiogenesis of maternal-fetal interface and the pathogenesis of PE (Ho et al., 2017; Eberlé et al., 2019; Ma et al., 2021), is directly modulated by GRK2 through receptor internalization and desensitization. We hypothesize there are potential links between roles of GRK2 and the onset of PE. However, insufficient research has evidenced the potential roles of GRK2 in preeclampsia until now.

In current study, we first aimed to identify the expression patterns of GRK2 in the placental vasculature of human patients and a murine model throughout gestation. We found that GRK2 expression was required by CTBs, STBs, and EVTs. Furthermore, downregulation of GRK2 was discovered in PE patients (*P* < 0.0001), suggesting a potential protective role of GRK2 against PE. Knockdown or inhibition of GRK2 stimulated dose-dependent activation of RIPK3 and MLKL *in vitro*, where nuclear envelope rupture and cellular DNA leakage were also found (Yoon et al., 2017; Zhang H. et al., 2020). It was hypothesized that GRK2 deficiency causes poor placentation; this was tested through the use of an *in vivo* murine model with knockdown or inhibition of placental *GRK2*. The tested mice exhibited overt placental thrombosis, infarction, and cavitary lesions, as well as hypertension and proteinuria in late gestation. These findings depict a cause-effect relationship between GRK2 deficiency and clinical syndromes. Further, an insignificant change in blood pressure and urinalysis results were observed in non-pregnant mice injected with the *GRK2* knockdown lentivirus. These findings could offer novel insights into the pathology of PE due to the proposed role of GRK2 as a critical protein in the prevention of trophoblast necroptosis and poor placentation. In a wider context, this research could facilitate drug development for therapeutic intervention of PE by modulating GRK2-mediated necroptosis.

## Materials and Methods

### Patient Recruitment and Human Placenta Sample Collection

Placentae at full term (37–41 weeks of gestation) were obtained from patients within 15 min of cesarean deliveries. The inclusion criteria and procedures for placenta collection were in accordance with those described previously (Chen et al., 2018). A total of 25 healthy, pregnant women and 26 patients with PE were enrolled in the study from September 2016 to July 2017. A positive diagnosis of PE was defined as systolic blood pressure ≥ 140 mmHg or diastolic blood pressure ≥ 90 mmHg during the last two blood pressure measurements after the 20th week of gestation, in the presence of significant proteinuria (≥ 300 mg/24 h), without preexisting renal disease or primary hypertension. The patients with PE did not have any other maternal complications; gestational hypertension or idiopathic IUGR should be excluded. The decidua basalis and chorionic plate were removed from the placentae, and the villous tissue was dissected and rinsed in cold physiological saline. For each placenta, tissue fragments were obtained from 4 “quadrants” in a north-south-east-west pattern to limit tissue heterogeneity. Biopsy specimens were taken from the maternal side of the placenta and rinsed in sterile phosphate buffered saline (PBS) solution to remove blood. The tissue samples were dissected in Petri dishes and liberated from the maternal vessels and tissue; the samples were then adjusted to 150 mg. Placental villous tissue was divided into 3 treatment groups: (1) snap-frozen in liquid nitrogen and stored at 80°C for proteomic analysis (2) 150 mg of tissue was mixed with 2 ml of a commercial RNA Keeper Tissue Stabilizer (CA#R501, Vazyme, Piscataway, NJ, United States), stored at + 4°C overnight and then moved to −80°C for long-term storage (3) tissues were fixed in 4% paraformaldehyde and either embedded in paraffin or cryopreserved in optimal cutting temperature (OCT) compound. The patients’ clinical features are characterized in Supplementary Table 1.

### Villous Explant Culture

Previously established methods were modified to culture villous explants (Newby et al., 2005). Villous tissue samples (*n* = 8) were collected from patients who had undergone legal termination, not due to virus infection or alike other medical reasons, during 6–13 weeks of gestational age. The exclusion criteria for the study included patients with a history of spontaneous abortion or ectopic pregnancy. Villous tissue was isolated, washed several times in sterile PBS, and carefully dissected into approximately 2 mm tissue explants. These samples were then placed in individual wells of a 24-well plate pre-coated with 150 μL solution containing 1 μg/μL BD Matrigel*™* Basement Membrane matrix (CA# 354234, BD Biosciences, San Jose, CA, United States) and incubated at 37°C for 4 h to ensure polymerization. Following this, the explants were cultured at 37°C, 5% oxygen, and 5% CO2 in DMEM/F12 (CA#1133003, Gibco, CA, United States) medium containing 10% fetal bovine serum (CA#SA112, FBS, CellMax, Beijing, China), 2 mM L-Glutamine (CA#G63192 Sigma-Aldrich, St. Louis, MO, United States), and 100 U/mL Penicillin-Streptomycin (CA# 15070063, Gibco, CA, United States). Explants that showed stable attachment and outgrowth on the gel matrix were recorded after 48 h, using inverted phase-contrast microscopy (EVOS FL Color Imaging System; Thermo Fisher Scientific). Explant cultures were washed with icy PBS solution for a minimum of 3 times, and separated into 3 groups: branches, tips (villous column tips), and EVT cells (the cells attached to the gel matrix). Tissue from each of the 3 groups was lysed in TRIzol reagent (CA#15596018, Takara, Tokyo, Japan) and synthesized first strand cDNA with the Prime Script RT reagent kit (CA# RR047A, Takara, Tokyo, Japan). Finally, the cDNA was stored for RT-qPCR analysis. The patients’ characteristics are displayed in Supplementary Table 2.

### Cell Culture

The immortalized first-trimester human trophoblast cell line HTR8/SVneo was purchased from the American Type Culture Collection (ATCC; Manassas, VA, United States). The cells were cultured in RPMI-1640 medium (CA#1263301, Gibco, CA, United States) supplemented with 10% FBS (CA#SA112, FBS, CellMax, Beijing, China) and incubated at 37°C, 5% CO2. The scrambled control siRNA (NC-siRNA) (5′-UUCUCCGAACGUGUCACGUTT-3′) and siRNA-GRK2 (5′-GAAGTACGAGAAGCTGGAGTT-3′) were synthesized by GenePharma Inc., (Shanghai, China), and transfected into the HTR-8/SVneo cells using EndoFectin*™* Max (CA#EF013, GeneCopoeia Inc., Rockville, MD, United States), according to the manufacturer’s instructions. The cells were cultured for 24 h after transfection and then treated with caspase inhibitor z-VAD-FMK (z-VAD; 25 μM; MCE, Monmouth Junction, NJ, United States) and necroptosis inhibitor necrostatin-1 (Nec-1; 30 μM; MCE, Monmouth Junction, NJ, United States). For the control group, the cells were cultured for 48 h after transfection, followed by direct examinations.

### Mice

Sixty female and 10 male ICR mice were obtained at 8–10 weeks weighing 25–30 g from the Vital River Laboratory Animal Technology Co., (Beijing, China) (mice certificates: No.1107272011003380 and No.1107272011001996). The mice were housed under specific pathogen-free (SPF) conditions with a 12-h light/12-h dark cycle at the Experimental Animal Center of Chongqing Medical University. Male mice were only used for breeding, whilst female mice were used for experimental purposes. Firstly, the female mice were mated with age-matched ICR male mice, then randomly divided into five groups (*n* = 6–10) including the control group, GRK2 inhibitor 2 mg/kg group, GRK2 inhibitor 4 mg/kg group, negative lentivirus group, and GRK2-KD lentivirus group. Vaginal plug detection was carried out on E0.5. The control group comprised age-matched, pregnant ICR mice without treatment of small-molecule compound or lentivirus. From E8.5 to E13.5, mice in the GRK2-inhibitor-treated group received daily intraperitoneal injections of 100 μl GSK180736A (2 or 4 mg/kg; MCE, Monmouth Junction, NJ, United States) diluted in corn oil. Mice in the lentivirus-treated group received intrauterine injections of GRK2-KD lentivirus (3 × 10^7^ *pfu*, 100 μl) under anesthesia using isoflurane once at E8.5. Lentivirus expressing shRNA targeting mice GRK2 lentiviral vectors (GV493) was purchased from GeneChem (Shanghai, China). The shRNA sequences are listed below: *GRK2: 5*′*-GAGATCTTTGACTCCTATATT-3*′. Mice in the NC-LV group received intrauterine injections of a negative-control virus (CON238, GeneChem, Shanghai, China) once at E8.5. Blood pressure was monitored every other day from E0.5 to E14.5, and daily from E14.5 to E18.5, using the tail-cuff photoplethysmography BP-2000 SERIES II (Visiotech System; Apex, NC, United States). On E16.5, the 24-h urine samples were collected using a metabolic cage. At E18.5, the mice were anesthetized with highly concentrated isoflurane in CO_2_ (∼60% inhalation) to allow blood, kidney, and placenta collection. Treatment outcomes comprised evaluation of PE symptoms on E18.5 of pregnancy, such as hypertension, proteinuria, circulating soluble fms-like tyrosine kinase 1 (sFlt1), and endothelin-1 (ET-1). Pregnancy outcome was assessed by uterine bleeding, murine fetal resorption, litter size, embryo weight, and placenta weight. Histological assessment included evaluation of placental and kidney biopsy specimens. Weights were measured using electronic balances (Mettler Toledo XP56-0.001 mg, Zurich, Switzerland), whilst lengths were measured by Vernier caliper. Samples of placental tissue were removed for protein and RNA analyses, as well as paraffin embedding. The remainder of the placental labyrinth tissues and kidney cortexes were cut and fixed in 3% glutaraldehyde. The non-pregnant control group were age-matched females, receiving intrauterine injections of GRK2-KD lentivirus (3 × 10^7^ *pfu*, 100 μl). All data from non-pregnant mice is shown in Supplementary Figures 3, 4.

### Immunohistochemistry Staining and Immunofluorescence

The placental tissue and kidney tissue samples were washed with PBS and fixed with 4% paraformaldehyde at room temperature overnight. After this period, the samples were dehydrated and embedded in paraffin or cryo-embedding media (OCT), before slicing into sections [Paraffin-embedded Tissue Sections for immunohistochemistry (IHC); OCT–embedded Frozen Tissue for immunofluorescence (IF)]. Staining kits, hematoxylin and eosin, periodic acid-Schiff, or von Kossa kits were purchased from Solarbio (Beijing, China). For immunohistochemical and immunofluorescent analyses, primary antibodies against von Willebrand factor (mouse, Santa Cruz, CA, United States), CD34 (rabbit, BIOSS, Beijing, China), cytokeratin 7 (rabbit, BIOSS, Beijing, China), proliferin-related protein (mouse antibody, Santa Cruz, CA, United States), placental lactogen-1 (mouse antibody, Santa Cruz, CA, United States), Cleaved-Caspase 3 (mouse antibody, Proteintech Corp., China), phospho-MLKL (Ser358) (mouse antibody, BIOSS, Beijing, China) were used to detect endothelial cells, the fragments of remodeled endothelium, trophoblast, trophoblast giant cells (TGCs), trophoblast giant cells (TGCs), activated apoptotic signaling and activated necroptotic signaling, respectively. Monoclonal GRK 2 antibodies were purchased from Santa Cruz Biotechnology (CA#sc-13143, mouse, Santa Cruz, CA, United States) and BIOSS Biotechnology (CA#bs-1209R, rabbit, BIOSS, Beijing, China). Secondary antibodies were conjugated with horseradish peroxidase, Anti-Rabbit IgG DyLight 488 (Green; Proteintech, Wuhan, China), or Anti-Mouse IgG Cy3 (Red; Proteintech, Wuhan, China). The tissue sections were mounted with or without DAPI-containing (blue) mounting medium (CA#AR1177, Boster, China). Control sections were treated without primary antibodies. A light or fluorescent microscope was used to take photographs using the EVOS FL Color Imaging System (Thermo Fisher Scientific, Waltham, MA, United States), or a fluorescence microscope was used (Olympus; VS200; Olympus Corporation, Tokyo, Japan). A minimum of five images were taken of each sample; all image quantifications were completed using Fiji Image J software.

### Transmission Electron Microscopy

HTR8/SVneo cells (1 × 10^7^) from each group were fixed in 3% glutaraldehyde overnight and embedded in epoxy resin. Placenta labyrinth tissue and kidney cortex samples from pregnant mice at E18.5 were fixed in 3% glutaraldehyde, then treated with 1% osmium tetroxide and embedded in an Araldite-Epon mixture (CA# 45345 Sigma-Aldrich, St. Louis, MO, United States). Semi-thin sections (0.6 mm) of each sample were prepared and examined with a transmission electron microscope (TEM) (JEOL JEM-1210) at Serviobio Corp., (Serviobio, Wuhan, China). A minimum of 3 images were taken for each sample.

### Reverse Transcription-Polymerase Chain Reaction

Total RNA was isolated from cultured cells, murine tissues, or human tissues using TRIzol reagent (CA#15596018, Takara, Tokyo, Japan). First-strand cDNA was synthesized using the Prime Script RT reagent kit (CA# RR047A, Takara, Tokyo, Japan). RT–PCR was achieved using oligonucleotide primers specific for human *GRK1*, *GRK2*, *GRK3*, *GRK4*, *GRK5*, *GRK6*, *GRK7*, or β*-actin* (Supplementary Table 3), and quantified mRNA levels shows on Supplementary Figure 1. β*-actin* was used as an internal control. Quantitative reverse transcription-polymerase chain reaction (RT–PCR) was carried out using the Bio-Rad CFX Connect Detection System (Bio-Rad, CA, United States).

### Western Blotting

Total protein was extracted from placental tissues using radio-immunoprecipitation assay buffer (CST, United States) containing phenylmethylsulfonyl fluoride (CST, United States). Protein was quantified using the BCA assay reagent (CA# P0010, Beyotime, Nanjing, China) according to the manufacturer’s protocol. After denaturation, equal amounts of protein extracts were resolved by 10 or 15% sodium dodecyl sulfate-polyacrylamide gel electrophoresis, and then transferred onto polyvinylidene difluoride membranes (Roche, Mannheim, Germany). The membranes were blocked with 5% skim milk for 2 h at room temperature, and then incubated overnight at 4°C with primary antibodies against GRK2 (1:1,000, mouse; Santa Cruz, CA, United States), CC3 (1:1,000, mouse; Proteintech Corp., China), Bax (1:1,000, rabbit; BIOSS, China), Bcl2 (1:1,000, mouse; Santa Cruz, CA, United States), RIPK1 (1:1,000, rabbit; BIOSS, China), RIPK3 (1:1,000, rabbit; BIOSS, China), phosphorylated MLKL (p-MLKL) at serine 358 (1:1,000, mouse; BIOSS, China), and β-actin (1:5,000, mice; Proteintech, China). HRP-conjugated goat anti-rabbit (1:5,000, goat; Abbkine, United States) and anti-mouse (1:5,000, goat, Abbkine, United States) secondary antibodies were used to detect the respective protein bands. Signals were quantified using the Image Lab software (Bio-Rad, Hercules, CA, United States).

### Coimmunoprecipitation and Immunoblotting Assays

The cells were collected as previously described in the western blotting section. Cells were lysed in binding buffer (50 mM Tris–Cl pH 7.5, 150 mM NaCl, 1 mM EDTA, 1% Triton X-100 and protease inhibitor). The monoclonal antibody against RIPK1(CA#sc-133102, mouse, Santa Cruz, CA, United States) or against RIPK3 (CA# sc-374639, mouse, Santa Cruz, CA, United States) or a matched-isotype antibody IgG (CST) was incubated with Protein A/G Magnetic Beads (MCE, Monmouth Junction, NJ, United States) at 4°C overnight. On the second day, a total of 1 mg protein sample lysate was incubated with an antibody-beads complex for more than 8 h. Then, the magnetic rack was used to precipitated the antibody-beads complexes. Then, the complexes were washed for three times and then eluted from beads by boiling at 95°C with 4 × LDS loading buffer (CA#161-0747, Biorad, United States). Finally, these protein samples were resolved by SDS/PAGE assay and immunoblotted with antibodies as indicated.

### Cell Viability Assays

Cell viability was determined 48 h after siRNA treatment. A total of 8 × 10^3^ HTR8/SVneo cells suspended in 100 μl cell cultural medium were added into each well of a 96-well plate and mixed with the Cell Counting Kit-8 (CCK-8; CA# HY-K0301, MedChemExpress, NJ, United States). This was followed by incubation at 37°C for 4 h. Absorbance was measured at 450 nm using the Multiskan Go microplate reader (Thermo Fisher Scientific, Waltham, MA, United States). 5-ethynyl-2′-deoxyuridine (EdU) staining was performed using the EdU kit (CA#C 10310-1, RiboBio, Guangzhou, China), according to the manufacturer’s instructions. EdU incorporation was visualized with the EVOS FL Color Imaging System (Thermo Fisher Scientific, Waltham, MA, United States). The EdU (magenta) and Hoechst (blue; CA#AR0039, Boster, China) staining channels were segmented; cells with overlapping staining were considered to be positive. Ki-67 is a nuclear cell proliferation-associated antigen expressed in all active stages of the cell cycle. Therefore, it was used to detect proliferating cells. Ki-67 signal was an artifact caused by the binding of mouse monoclonal Ki-67 antibody (1:100, mouse; Santa Cruz, CA, United States) to a secondary Cy3- conjugated goat anti-mouse antibody (1:100, Red; Proteintech, Wuhan, China). Image quantification was conducted using the Fiji Image J software.

### Analyses of Apoptosis and Necroptosis

Terminal deoxynucleotidyl transferase dUTP nick end labeling (TUNEL) was performed in OCT-embedded human placental sections and paraffin-embedded murine placental sections, using the Roche *in situ* fluorescein detection kit (CA#11684795910, Roche, Basel, Switzerland), in accordance with the manufacturer’s instructions.

### Flow Cytometric Analysis

Annexin V-FITC/PI Apoptosis kit (CA#KGA103, KeyGen Biotech Co., Nanjing, China) was used to determine phosphatidylserine and membrane integrity of the samples. Cells were stained with PI and Annexin V FITC simultaneously prior to flow cytometric analysis (BD LSRFortessa, United States). PI-positive cells were designated end stage apoptotic and necrosis cells, while FITC-positive cells were designated early stage apoptotic cells.

To determine mitochondrial membrane potential, HTR8/SVneo (5 × 10^5^) cells were seeded in 6-well plates for 24 h prior to quercetin treatment (40 μM). Positive and negative substrates in the JC-1 Mitochondrial Membrane Potential Detection Kit (CA#2006, Beyotime, Nanjing, China) were added and incubated at 37°C for 20 min. Cells were then washed twice with 1 × PBS and finally resuspended in 500 μL PBS. Potential dependent accumulation in the mitochondria is shown by the JC-1 dye as a fluorescence shift from green to red, suggesting early apoptosis. Measurements were analyzed on a flow cytometry (BD LSRFortessa, United States), using a 488 nm excitation laser in combination with FITC and PE emission channels to measure green and red fluorescence of the JC-1 stain, respectively. Results were analyzed using the FlowJo^®^ software (FlowJo LLC, Ashland, OR, United States).

### Matrigel Invasion Assay

The invasion assay was performed in a transwell chamber consisting of a 24-well plate with membrane inserts (CAT#CLS3524, Corning Costar, Cambridge, MA, United States) containing polycarbonate transwell filters (8-μm pore size, Corning Costar, Cambridge, MA, United States) precoated with 60 μl of 1 mg/ml Matrigel Basement Membrane Matrix solution (CAT#356234, BD Biosciences, San Jose, CA, United States). Approximately 8 × 10^4^ cells in 200 μl serum-free culture medium was seeded into the upper chamber, and each insert was placed in the lower chamber containing 600 μl RPMI1640 medium containing 10% fetal bovine serum. After incubating for 24 h, the migrated cells were fixed by 4% paraformaldehyde and stained by crystal violet. Next, cells that penetrated the membrane were observed under a light microscope (EVOS FL Color Imaging System, Thermo Fisher Scientific, Waltham, MA, United States) and the number of cells from five fields was analyzed at random using Fiji Image J software. Notably, Cell was collected, counted and re-seeded at 24 h post-transfection or 24 h after pharmacological exposure. All experiments were repeated in triplicate.

### Wound-Healing Assay

HTR8/SVneo cells (1.2 × 10^5^cells/well) were seeded onto 6-well plates and grown to more than 90% confluence, and then a scratch on the cell monolayer was made using a 200 μl pipette tip. The cells were then rinsed twice with fresh culture medium and 2 ml cell culture medium were added to each plate, and allowed to recover for 24 h, and pictures were taken at 0, 12, and 24 h. The area of wound healing was measured and analysis with Fiji Image J software. Notably, Cell was collected, counted and re-seeded at 24 h post-transfection or 24 h after pharmacological exposure. Each experiment was performed in triplicate.

### Enzyme-Linked Immunosorbent Assay

Blood samples were taken from mice at E18.5 in serum gel tubes and allowed to clot for 45 min. Samples were then centrifuged at 1,320 × *g* for 10 min at room temperature. Supernatant was transferred to separate 2 ml Protein LoBind^®^ Tubes (Eppendorf, Hamburg, Germany) and stored at −80°C prior to analysis. Serum soluble fms-like tyrosine kinase 1 (sFlt-1) and endothelin-1 (ET-1) levels were determined using the commercial ELISA kit sVEGFR-1 (SEB818Mu; Cloud Clone Corp., Houston, TX, United States) and EDN1 ELISA Kits (CEA482Mu; Cloud Clone Corp., Houston, TX, United States), respectively, according to the manufacturer’s instructions. Urine was collected at E16.5 by metabolism cage and transferred to a sterile collection container. Centrifugation was undertaken for 10 min at 2,000 × *g* at room temperature. The top 80% Of each sample was transferred to separate fresh tubes followed by storage at −80°C. Urinary albumin was measured using the commercial ELISA Kit: Albumin ELISA Kit (CEB028Mu; Cloud Clone Corp., Houston, TX, United States), according to the manufacturer’s instructions. Plate absorbance was read at 450 nm by the Multiskan Go microplate reader (Thermo Fisher Scientific, Waltham, MA, United States). The concentrations of sFlt-1, ET-1, and albumin were calculated using a four parametric logistic standard curve with ELISAcalc^®^ software *V1.0.*

### Statistical Analysis

**FIGURE 1:**
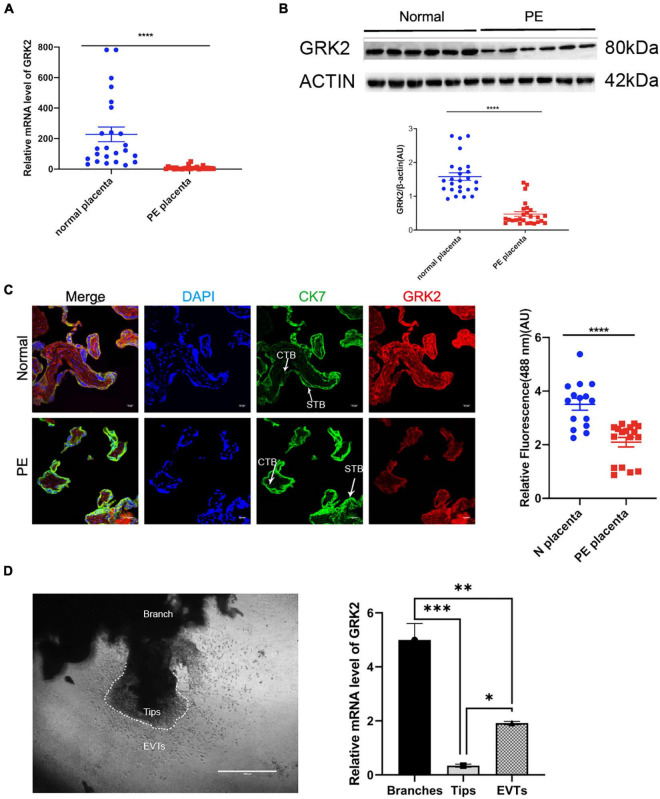
Reduced expression of GRK2 characterizes the preeclamptic placenta. **(A)** GRK2 mRNA levels were determined in the term placenta samples by qRT-PCR. Each dot is representative of a patient sample. **(B)** Western blotting revealed the protein levels of placental GRK2. Each dot is representative of a patient sample. **(C)** Immunofluorescence co-staining and quantification of GRK2 (red), CK7 (green; trophoblasts), and DAPI (blue; nuclei) in term placentae. Scale bar, 100 μm. **(D)** qRT-PCR analysis of GRK2 mRNA abundance in the first-trimester villous explants (*n* = 8). The tips, branches, and EVT outgrowth cells were isolated along the villus explants on Matrigel-coated wells at 48 h post-explant. Data are shown as the mean ± SEM. PE, preeclampsia; GRK, G Protein-Coupled Receptor Kinase; CTB, cytotrophoblast; STB, syncytiotrophoblast; EVT, extravillous cytotrophoblasts. *P < 0.05, ^**^P < 0.01, ^***^P < 0.001; ****P < 0.0001.

The GraphPad Prism 8 software (GraphPad software, San Diego, CA, United States) was used for data analysis. Results were expressed as mean ± standard error of the mean (SEM), unless otherwise stated. The gestational age and proteinuria in Supplementary Table 1 were analyzed by Mann–Whitney *U*-test. Statistics from Figures 1–C were analyzed by student’s *t*-test. Statistics significance in other figures was assessed by one- or two-way analysis of variance (ANOVA). If significance was identified, post hoc analysis was undertaken using Tukey’s HSD test for specific comparisons between two groups. Results were deemed as significant when *P* < 0.05.

## Results

### Reduced Expression of GRK2 Characterizes the Preeclamptic Placenta, While Its Expression Is Indispensable for EVTs

To investigate the potential role of GRKs in the pathology of PE, we evaluated the GRK subtypes in end-stage placentae of healthy patients (control group) and PE patients, where we first discovered the down-regulated GRK2 levels in PE placentae in both mRNA and protein levels, compared to controls (Figures 1A,B and Supplementary Figure 1). The GRK2 mRNA was highly expressed in the normotensive group, yet it was downregulated in the PE group (1.528 vs. 0.6509, *P* < 0.0001; unpaired *t*-test); the protein levels in PE placentae were also down-regulated by over 50% (*P* < 0.0001, unpaired *t*-test). The red signaling fluorescence was also shown to have reduced by 41% in the PE group (1.528 versus 0.6509, *P* < 0.0001; unpaired *t*-test); these findings suggested that GRK2 might be involved in the pathogenesis of PE (Figures 1A–C). Further, immunofluorescence images showed extensive and stable expression of GRK2 in the cytoplasm of cell column trophoblasts throughout gestation (Figure 1C and Supplementary Figure 2). This Spatio-temporal pattern localization in villi was further determined in wide type (WT) pregnant mice. Besides, the use of chorionic villous explants cultured on Matrigel identified that the invasive EVTs and branched villi expressed considerable levels of GRK2 at early pregnancy (Figure 1D). This implies that GRK2 expression is necessary for extravillous and villous column trophoblasts. Collectively, the data strongly implicates the involvement of GRK2 in the pathogenesis of PE.

### Knockdown or Inhibition of GRK2 in the Placenta Induces a PE-Like Phenotype

**FIGURE 2:**
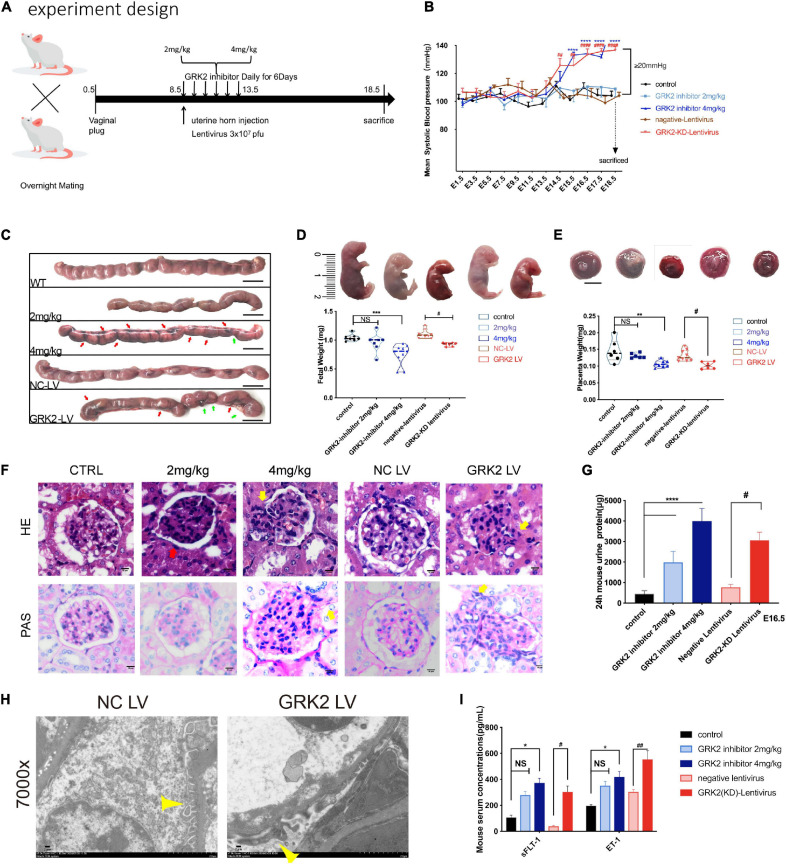
Knockdown or inhibition of GRK2 in placenta induces PE-like phenotype:hypertension, proteinuria, and angiogenic imbalance in mice. **(A)** Experimental design. For each group, *n* = 6–10 mice. **(B)** Blood pressure was taken in mice with/without placental knockdown or inhibition of GRK2. *n* > 6, Mean ± SEM. **(C)** Gross morphology of uterus at E18.5 shows the spontaneous hemorrhage and resorptions of embryos in uteri of GRK2 deficient groups. Green arrows, fetal resorption; red arrows, intrauterine hemorrhage. **(D,E)** Knockdown or inhibition of placental GRK2 induced embryo growth restriction and placenta growth restriction. **(F,G)** Glomerular histopathology assessment by HE (upper panel) and PAS (lower panel) staining of untreated mice and mice treated with GSK180736A (2 or 4 mg/kg), and mice receiving intrauterine injections with non-target/GRK2-knockdown lentivirus. Scale bar, 10 μm. Mesangial hyperplasia (yellow arrow) and decreased capillary density (red arrow) are shown in GRK2-knockdown/inhibition group. **(H)** TEM analysis of glomerulus. Pathological podocyte fusion in GRK2-knockdown group and the podocytes in NC-LV group are indicated by thin yellow arrows; 7,000× magnification, Scale bar, 2 μm. **(I)** the sFlt1 and ET-1 abundance in serum of E18.5 mice, as determined by ELISA. sFlt1, soluble fms-related tyrosine kinase-1; ET-1, Endothelin-1. All data are represented as X ± SEM from each experiment (*n* = 6–10 mice, each assayed individually), NS, not significant; *P < 0.05, **P < 0.01, ***P < 0.001, ****P < 0.0001 versus control mice group; ^#^*P* < 0.05, ^##^*P* < 0.01, ^###^*P* < 0.001, ^####^*P* < 0.0001 versus NC-LV mice group.

To see if the down-regulation of trophoblastic GRK2 levels was intimately associated with the pathogenesis of preeclampsia, we set up experiments on pregnant mice. We divided 60 pregnant mice into 5 sub-groups (Figure 2A): normal pregnancy (CTRL, also labeled as WT); two groups receiving intraperitoneal injections of GRK2 inhibitor, GSK180736A at 2 mg/kg or 4 mg/kg daily from the 8.5-day embryo (E8.5) to 13.5-day embryo (E13.5); two groups receiving intrauterine injections of negative (NC-LV) or GRK2-KD lentivirus (3 × 10^7^ *pfu*) (GRK2-LV) once on E8.5 (KD efficiency shown on Supplementary Figure 6). The non-pregnant female mice receiving intrauterine injection of GRK2-KD lentivirus (*3* × *10^7^ pfu*) were used as the blank control (Supplementary Figures 3, 4). Mice from the 4 mg/kg and GRK2-LV group developed elevated blood pressure from E14.5 to E18.5 (had surged over 20 mmHg compared to early pregnancies), which closely resembled the late gestational hypertension in pre-eclamptic women (Figure 2B). These non-pregnant females intrauterine injected with the same dose of GRK2-KD lentivirus and continuously monitored on blood pressure for 10 days, displayed no significant pressure elevations (Supplementary Figure 3A). We performed cesarean sections at E18.5 (near birth), when we found abnormal hemorrhage, clots in uteri, and resorptions of embryos in embryos from 4 mg/kg and GRK2-LV groups (Figure 2C). These obscure bleeding and stillbirth have been widely reported in PE patients (Gibbins et al., 2016; Gardiner and Vatish, 2017). Accordingly, the placental deficiency of GRK2 resulted in smaller placentae and lighter pup masses, yet there was no difference in litter size (not shown in figures) (Figures 2D,E). Proteinuria and renal dysfunction are main hallmarks of preeclampsia. Hence, we tested mice 24 h urinary proteins before mating and at late gestational age. Though similar urinary protein levels between groups before pregnancy, the urinary protein levels increased positively correlated to the extent of GRK2 deficiency in a dose-dependent manner at E16.5 (Figure 2G), consistent with their swelled glomerular tufts and reduced Bowman’s spaces. Periodic acid–Schiff staining further revealed enhanced extracellular matrixes accumulation, accelerated mesangial expansion and collapsed glomerular capillaries in 4 mg/kg and GRK2-LV groups (Figure 2F). Random transmission electron microscopy (TEM) samples of the glomeruli were used to measure the thickness of glomerular basement membrane (GBM) and podocytes fusion in glomerular filtration barriers (Figure 2H; Hennessy and Makris, 2011). Additional typical features such as increased plasma soluble fms-like tyrosine kinase-1 (sFlt-1) and endothelin-1 (ET-1) levels were also observed elevated in 4 mg/kg and GRK2-LV group (Figure 2I), though these plasma cytokines were not elevated in non-pregnant mice with GRK2-LV intrauterine injection (Supplementary Figure 4) (Makris et al., 2007; Verdonk et al., 2015).

Together, these results indicate the knockdown or inhibition of placental GRK2 were sufficient for the development of PE-like clinical features.

### Knockdown or Inhibition of GRK2 in the Placenta Promotes Necroptosis, Consistent With Features of Preeclamptic Placenta

**FIGURE 3:**
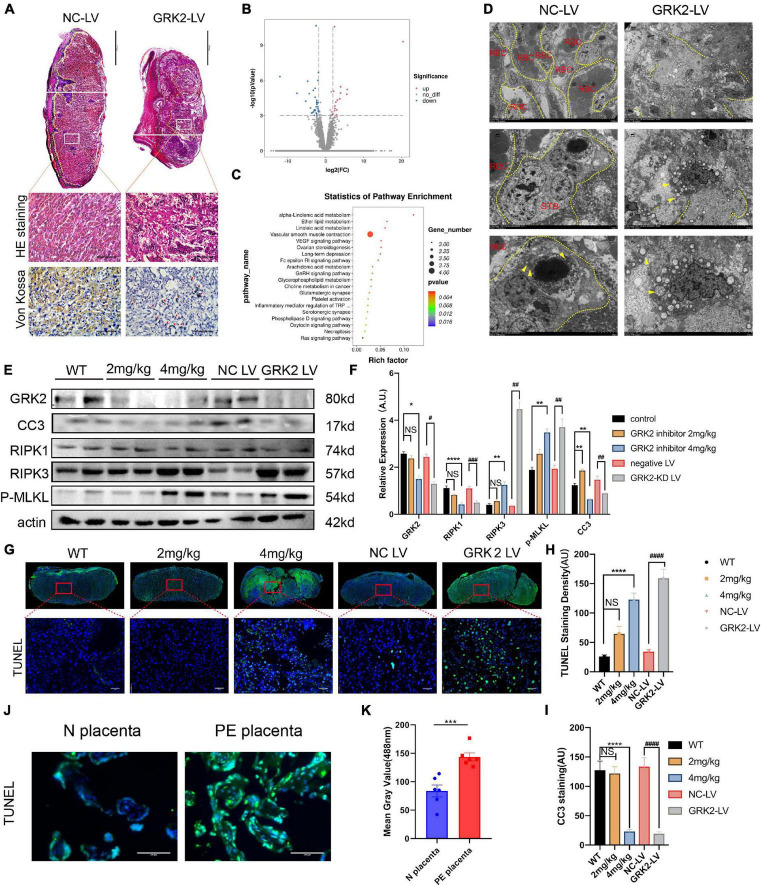
Suppression of GRK2 in placenta compromises placentation *via* necroptosis. **(A)** The 1st panel: Representative H&E staining images of E18.5 placentae from NC-LV and GRK2-LV groups. GRK2-LV placentae display a lack of vascularization, smaller placental size and a smaller labyrinth. Scale bar, 500 μm. Yellow lines, labyrinth and junction zone boundary; Green lines, junction zone and decidua boundary. The 2nd panel: Higher magnification of the upper white rectangle. Scale bar, 100 μm. 2nd and 3rd panels: The H&E staining and Von Kossa staining illustrate placental infarction and calcium deposition (red arrows) in GRK2-knockdown mice, respectively. **(B)** Volcano plots show differential expression for comparisons between GRK2-LV and NC-LV placentae (*N* = 3 per group. **(C)** Kyoto Encyclopedia of Genes and Genomes (KEGG) pathway analysis for the differentially expressed genes in total RNA-seq: GRK2-LV vs. NC-LV groups (*N* = 3 per group). **(D)** TEM images show extensive necrosis in labyrinth layer of GRK2-LV group. Yellow dotted line, cell membranes; Yellow arrows swelling mitochondria. From upper to bottom, 500×, 1,500×, 2,500× magnification, respectively. **(E,F)** Western blot analysis of placental cleaved-caspase 3, RIPK1, RIPK 3, phospho-MLKL (p-MLKL; S358), and β-actin (loading control). The DNA fragment analysis **(G,H)** and apoptosis rate analysis **(I)** in mice term placentae. Green signal indicates TUNEL-positive nuclei. Scale bar, 50 μm. **(J,K)** The DNA fragment in patients’ placentae. Scale bar, 100 μm. Dec, decidua; JZ, Junction Zone; Lab, labyrinth; H&E, hematoxylin and eosin staining; RBC, red blood cells; STB, syncytiotrophoblast. Data present as X ± SEM; NS, not significant; *P < 0.05, **P < 0.01, ***P < 0.001, ****P < 0.0001 versus WT group; ^#^*P* < 0.05, ^##^*P* < 0.01, ^###^*P* < 0.001, ^####^*P* < 0.0001 versus NC-LV group.

To analyses histopathological changes behind utterly distant gross morphology, we stained embryos with hematoxylin, eosin, or Von kossa (Figure 3A). The knockdown of GRK2 in placentae resulted in sterile necrotic lesions and calcium deposits in the placental labyrinth. We sequenced total RNA from placentae harvested at E18.5. Total RNA revealed up-regulated genes (*n* = 497), and down-regulated genes (*n* = 556) in placentae between NC-LV and GRK-LV groups (Figure 3B). The Kyoto Encyclopedia of Genes and Genomes (KEGG) pathway enrichment analysis with a fold change more remarkable than three revealed that the differentially expressed genes were mainly enriched in pathways related to “vascular smooth muscle contraction,” “hormone biosynthesis,” and “inflammatory mediator regulation.” We compared the differentially activated pathways in total RNA-seq and determined an up-regulated category, “necroptosis signaling,” as a potential critical pathway that might activate the downstream inflammation, oxidative stress, and vascular smooth muscle contraction in placentae (Figure 3C). Then we determined the presence of placental necrosis in the GRK2-LV group by TEM, where showed extensive damage of cell integrity, devoid of cell-cell adhesion, and swelling of mitochondria (Figure 3D, right panels). In contrast, placentae in the NC-LV group presented typically shaped nuclei, healthy organelles, and clearly outlined cell boundaries (Figure 3D, left panels). These necrotic features were further validated by western blot analysis (Figures 3E,F). Notably, the levels of GRK2 in placentae did not down-regulated significantly in the 2 mg/kg group (Figures 3E,F), which may explain a few necrotic lesions in histopathological specimens (Figures 3G,H).

Based on previous findings, we hypothesized that trophoblasts with GRK2 deficiency might activate necroptosis and exceed trophoblasts necrotic chains, retain the density of trophoblast giant cells, then lead to impaired spiral artery remodeling, and further result in placental mal-perfusion and hypertension at late gestation. To verify this conjecture, we used TUNEL staining for detecting DNA fragmentation in necrosis and cleaved caspase-3 immunocytochemical staining to detect the caspase-dependent apoptosis (Figures 3G,I). Significantly activation of necrosis was detected in the 4 mg/kg group and GRK2-LV group (Figures 3A–F), while the observed necrosis was not caspase-dependent (*P* < 0.0001) (Figures 3G–1). Meanwhile, DNA fragmentation in villous column and necrotic features surrounding spiral arteries were detected in placentae from patients (*P* < 0.001) (Figures 3J,K and Supplementary Figure 6C). Thus, we hypothesized that GRK2 levels in the placenta is inversely correlated with the activation of necroptotic events in placentae and with the severity of PE-like symptoms *in vivo*, in a dose-dependent manner.

### Knockdown or Inhibition of GRK2 Impaired Trophoblast Invasion and Spiral Artery Remodeling in the Placenta

**FIGURE 4:**
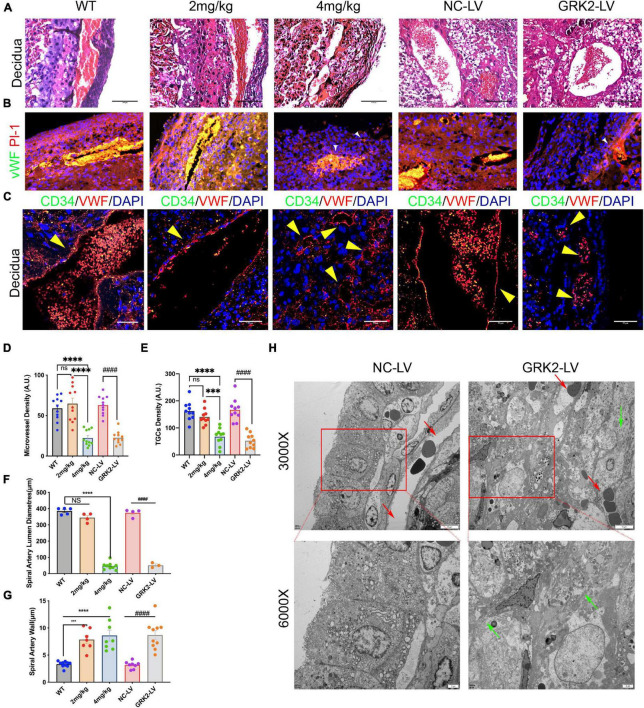
Suppression of GRK2 in placenta induces para-vascular necrosis and failures of spiral arterial remodeling. **(A)** E18.5 embryo decidual sections were stained with H&E. panel. Scale bar, 100 μm **(B)** Microvessels and trophoblast giant cells (TGCs) at E18.5 decidual-placental segments evaluated by vWF (endothelium, red) and Pl-1(parietal TGCs, green), respectively. White arrows (small vessels in GRK2-deficient group). vWF, von Willebrand factor; Pl-1, placental lactogen-1. Scale bar, 50 μm **(C)** Co-staining of CD34 (green signals), vWF (red signals), and nuclei to detect the microvessel parameters of E18.5 embryos. Yellow arrows indicate spiral arterial wall. Scale bar, 50 μm. **(D,E)** Quantity analysis of decidual microvessels and TGCs. **(F,G)** Morphometric analysis of spiral arteries. **(H)** The TEM images show impaired arterial remodeling with para-vascular necrotic lesions and cells. Red arrows, spiral artery; Green arrows, fragment membrane. From upper panel to bottom, 3,000×, 6,000× magnification, respectively. The ultrastructure of spiral arteries in GRK2-LV group was distorted, and its vascular lumen was almost closed. NS, not significant; ^***^P < 0.001, ^****^P < 0.0001 versus WT group; ^####^*P* < 0.0001 for NC-LV group.

Shallow invasion and insufficient spiral artery remodeling are expected in PE. As we had obtained the invasive EVTs expressed considerable levels of GRK2 mRNA (Figure 1D), we need ascertain whether EVTs necrosis may lead to failures in the spiral artery remodeling. The H&E stained specimens on Figure 4A showed much larger and more abundant decidual spiral arteries in WT/NC-LV groups. To further evaluate vascular pathology at choriodecidual interface, the endothelium was stained by ubiquitous endothelial marker Willebrand factor (vWF) or CD34 to analyze the development of microvessels (Figure 4B and Supplementary Figure 6) (Hecht et al., 2016); trophoblast giant cells (TGCs) were stained by TGC marker placental lactogen-1 (Pl-1) or proliferin-related protein (Plfr) (Figure 4 and Supplementary Figure 6; Simmons et al., 2007; Kibschull et al., 2014). It was observed that, in comparison with WT group, the severity of late-gestational hypertension and proteinuria correlated inversely to micro-vessel density at decidual segments. There was smaller arterial lumens, reduced micro-vessel densities, and thickened artery wall s in 4 mg/kg group and the GRK2-LV groups (*P* < 0.0001) compared to WT or NC-LV groups (Figures 4C–G). Next, we examined the decidual vessels by TEM (Figure 4H and Supplementary Figure 6), where these hypertension females presented with perivascular necrotic cells and distorted arterial lesions compared to these normotensive controls (Fosheim et al., 2019; Staff et al., 2020). Altogether, placental deficiency of GRK2 induced para-spiral arteries necroptosis, reduced the numbers of TGCs at the decidual-placental interface, and subsequently altered the decidual angiogenesis and correlated to systematically features.

### Knockdown or Inhibition of GRK2 Induces RIPK1/RIPK3-Dependent Necroptosis, but Does Not Affect Caspase-Dependent Apoptosis in HTR8/SVneo Cells

**FIGURE 5:**
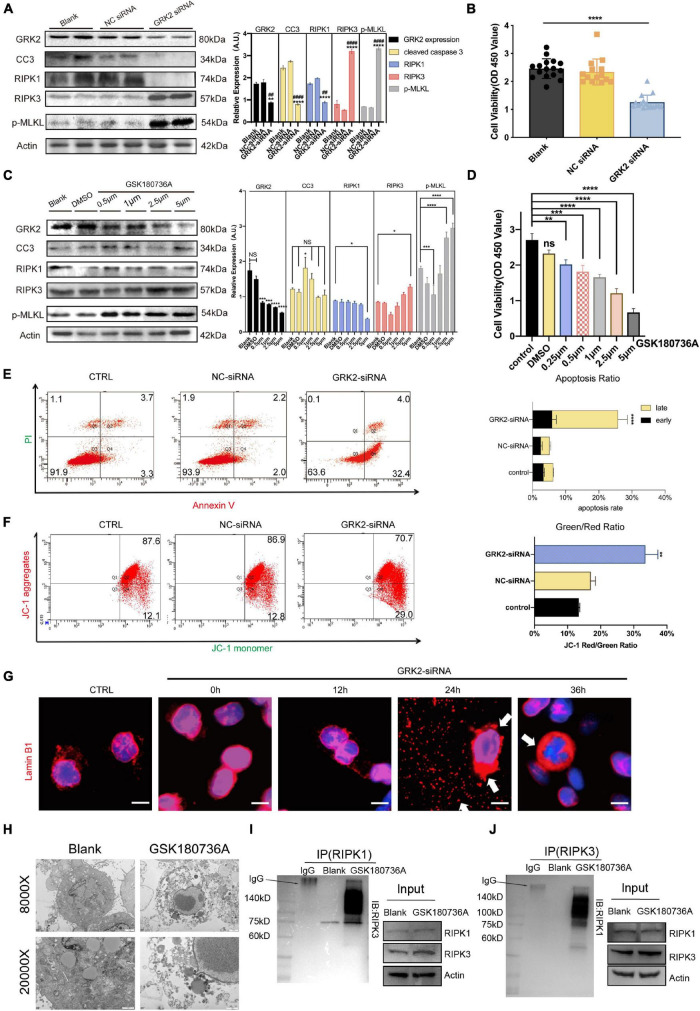
Knockdown or inhibition of GRK2 induces RIPK1/RIPK3-dependent necroptosis in HTR8/SVneo cells. **(A)** HTR8/SVneo cells are transfected with non-targeted siRNA (NC-siRNA) or siRNA targeted to GRK2 (GRK2-siRNA) for 48 h. Representative western blot analysis of cleaved-caspase 3, RIPK1, RIPK 3, phospho-MLKL (p-MLKL; S358), and β-actin (loading control). **(B)** Cell viability is assessed using CCK8 assay after transfection with siRNA for 48 h. Values are expressed as Mean ± SEM. **(C)** Pharmacologic inhibition of GRK2 in HTR8/SVneo cells by GSK180736A at different concentrations (0.5, 1, 2.5, and 5 μM) for 12 h. Representative western blot analysis of cleaved-caspase 3, RIPK1, RIPK 3, phospho-MLKL (p-MLKL; S358), and β-actin (loading control). **(D)** Cell viability is assessed using CCK8 assay after administration with GSK180736A for 12 h. Values are expressed as Mean ± SEM. **(E)** Flow cytometry with Annexin V/PI-combined staining shows early apoptosis (Ann V + /PI−), and late apoptosis/necrosis (Ann V + /PI +). The rate of late apoptotic/necrotic cells (Ann V + /PI +) is increased significantly after knockdown by GRK2-siRNA, ***P < 0.001. **(F)** To re-validate the necrosis rate induced by siRNA treatment, loss of mitochondrial membrane potential (ΔΨm) is measured by flow cytometry using JC-1 mitochondrial probes. **(G)** HTR8/SVneo cells transfected with GRK2-siRNA were examined for nuclear envelope integrity using Lamin B1 antibodies (red; nuclear envelope), or Hoechst (blue; nuclear context), at the indicated time points. Arrows indicate envelope breaches (Lamin B1 staining) and nuclear DNA leakage (Hoechst). Scale bar, 10 μM. **(H)** TEM images of EVTs treated with/without GSK180736A (cells from villi explants). EVTs on matrigel were necrotic after administration with GSK180736A. From upper to bottom: 8,000×, 20,000× magnification. **(I,J)** HTR8/SVneo cells treated with/without GSK180736A were collected and subjected to coimmunoprecipitation (Co-IP) with anti-RIPK1 antibody and anti-RIPK3 antibody. NS, not significant; *P < 0.05, **P < 0.01, ***P < 0.001, ****P < 0.0001 versus blank group; ^##^*P* < 0.01, ^####^*P* < 0.0001 versus NC-siRNA group.

To investigate the underlining mechanism, we used Small-interfering RNA (GRK2-siRNA) successfully silence the expression of GRK2 in HTR8/SVneo cells by approximately 51% after 48 h, which then triggered RIPK1/RIPK3-dependent necroptosis, without the activation of caspase-dependent apoptosis (Figure 5A). Cell viability assays showed an increased ratio of dead cells in the *GRK2-*knockdown (*GRK2*-KD) group (Figure 5B). To re-assess the non-apoptotic mechanism induced by *GRK2*-KD, GRK2 was pharmacologically inhibited by using GSK180736A at 0.5, 1, 2.5, and 5 μM for 12 h. We found that the high doses of GSK180736A induced dose-dependent activation of RIPK1/RIPK3/MLKL-dependent necroptosis, while a lower dose (0.5 μM) of GSK180736A exclusively activated typical caspase-dependent apoptosis (Figures 5C,D; Walsh et al., 2008; Moujalled et al., 2013; Oberst, 2016). Furthermore, the living, dead, early and late apoptotic/necrotic cells were quantified using flow cytometry with classical Annexin V/Propidium Iodide (PI) staining (Figure 5E), suggesting the *GRK2*-KD trophoblasts had a much shorter lifespan and accumulated necrotic cells. Also, the mitochondrial membrane potential JC-1 assay showed apoptotic and necrotic cells with changes in color of dye from red fluorescent to green fluorescent (Figure 5F), and indicated a similar ratio of necrotic cells in the *GRK2-*knockdown group (Chazotte, 2011; Crowley et al., 2016). Therefore, we determined the approximately 30% of cells were necrotic after 48 h followed by transfection, and almost no cells were apoptotic (less than 4%). We reasoned that the RIPK1/RIPK3-dependent necroptosis could be the dominant cell death mode in *GRK2*-KD trophoblasts, which may explain the significantly reduced cell viability in GRK2 deficient cells. To verify this, we stained HTR8/SVneo cells with an anti-laminin (red) antibody and nuclear marker DAPI (blue), as shown in Figure 5G. Then we found that at 24 h after transfection, in a considerable number of cells, the nuclear lamina ruptured from inside and nuclear envelope debris scattered around, while, at about 36 h after transfection, only a few pyknotic/fragmented nuclei were observed which might be the apoptotic cells (Figure 5G; Zhang T. et al., 2020). This was consistent with the short lifespan in the GRK2-siRNA group, which has been observed by flow cytometry. TEM images determined the typical necrotic ultra-structure of EVTs, cultured on matrigel of human villous explants and then administrated with GSK180736A for 24 h (Figure 5H). Furthermore, co-immunoprecipitation experiments were performed to determine necrosome formation after HTR8/SVneo cells were treated with GSK180736A. The immunoprecipitation products validated that the necrosome was pulled down only in HTR8/SVneo cells treated with GSK180736A, but not the control group (Figures 5I,J). Overall, the findings indicate that the knockdown or inhibition of GRK2 in HTR8/SVneo cells initiate RIPK1/RIPK3-driven necroptosis, which may be set as the cause of pathogenic consequences.

### Necrostatin-1 Rescues GRK2-Knockdown Trophoblasts From Nuclear Rupture and DNA Leakage

**FIGURE 6:**
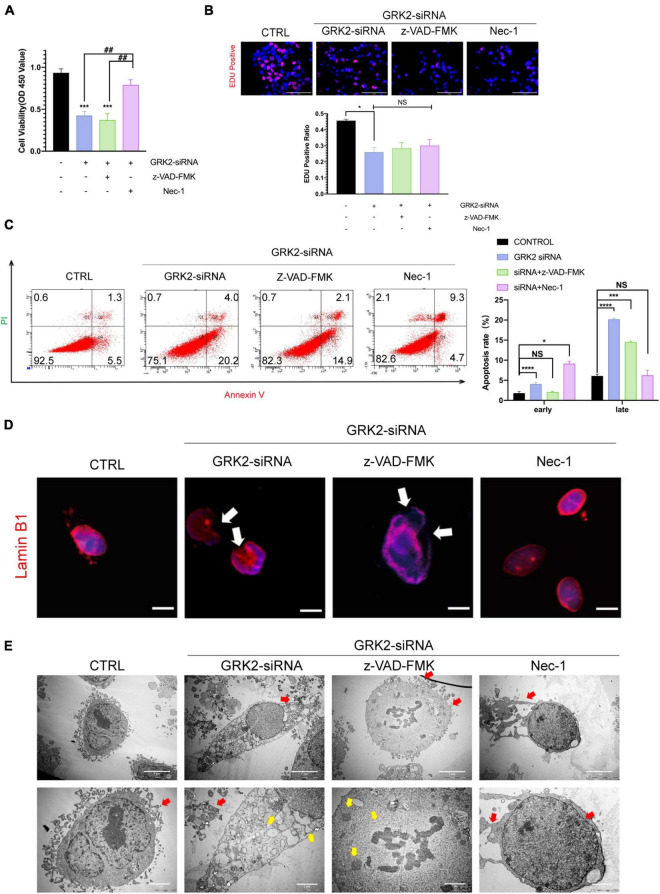
Suppression of Necroptosis rescues nuclear rupture and DNA leakage induced by reduced GRK2 levels in HTR8/SVneo cells. **(A)** Treatment of necrostatin-1 (Nec-1) enhances cell viability in GRK2 knockdown HTR8/SVneo cells, while cells treated with z-VAD-FMK cannot improve survival rates. **(B)** EdU (magenta) and DAPI (blue) staining images for cell proliferation analysis in HTR8/SVneo cells. The proliferation rates of cells transfected with GRK2-siRNA remain the same administration with/without z-VAD-FMK (25 μM, 12 h) or nec-1 (30 μM, 12 h). Scale bar, 100 μm. **(C)** Combined Annexin-V and PI staining was used to distinguish early apoptotic (Annexin-V + /PI−) and late apoptotic/necrotic cells (Annexin-V + /PI +). **(D)** Microscopy images demonstrated considerable nuclear deformation mediated by GRK2 knockdown. White arrows indicate distorted or ruptured sites of nuclear envelope. Scale bar, 10 μM. **(E)** Transmission electron microscopy (TEM) images show nuclear envelope breakdown (yellow arrow) and cell content outflow (red arrow) in HTR8/SVneo cells. The upper to bottom, 2,000× and 5,000× magnification, respectively. NS, not significant; *P < 0.05, ***P < 0.001, and ****P < 0.0001 versus control group; ^##^*P* < 0.01 versus GRK2-siRNA group.

Previous data has shown that *GRK2*-knockdown triggered HTR8/SVneo cells to excessive necroptosis *in vitro*. Therefore, we administrated caspase inhibitor z-VAD-FMK (z-VAD; 25 μM) or the necroptosis inhibitor necrostatin-1 (Nec-1; 30 μM) to *GRK2-*knockdown cells. We found that Nec-1 could restore cell viability in the absence of GRK2, unlike z-VAD-FMK (Figure 6A). Notably, the restored cell viability may be related to the reduced necroptosis than the increased ability of proliferation (Figure 6B). Also, by flow cytometry, we validated the reduced rate of necrosis in Nec-1 treated HTR8/SVneo cells, compared to cells only transfected with GRK2-siRNA (Figure 6C). Moreover, Nec-1 protected the nuclear morphology and nuclear envelope integrity, while the z-VAD-FMK did not (Figure 6D). TEM images showed the typical necrotic ultrastructural characteristics of *GRK2-*knockdown HTR8/SVneo cells (Figure 6E). These results validated that RIPK1/RIPK3-dependent necroptosis is the prioritized cell death pattern in GRK2-deficient trophoblasts, which Nec-1 can inhibit. The absence of GRK2 in trophoblasts, could initiate trophoblast necroptosis, attenuate transformation of spiral arteries, and lead to late-gestational hypertension and proteinuria.

### Knockdown or Inhibition of GRK2 Attenuates the Invasion and Migration Capability in HTR8/SVneo Cells After Excluding Dead Cells

**FIGURE 7:**
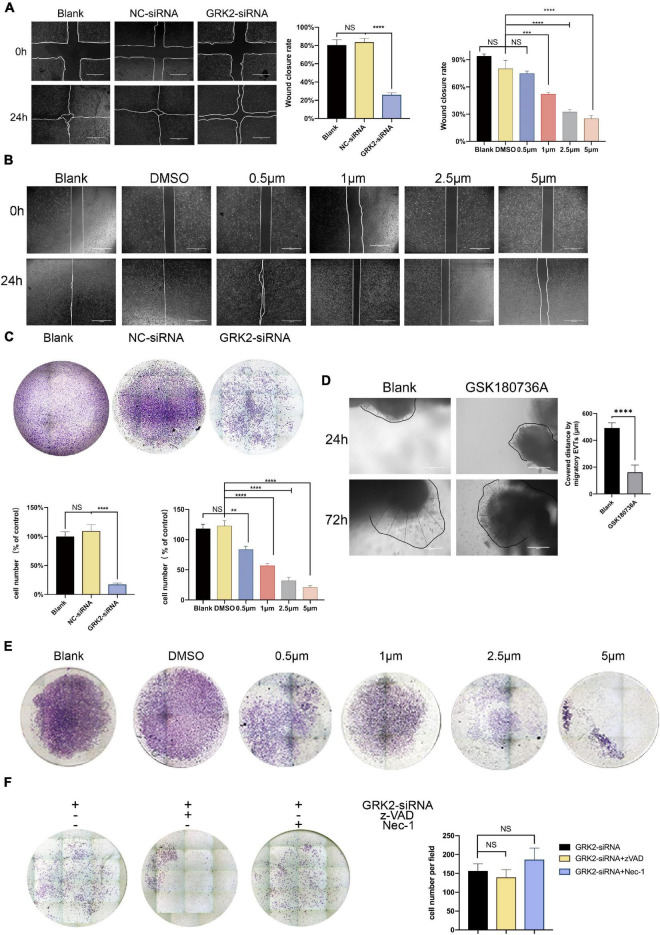
Decreased GRK2 levels or activity undermines migration and invasion in HTR8/SVneo cells, after excluding dead cells. **(A)** Migration ability of HTR8/SVneo cells assessed by wound closure assay. After 24 h, the wound edges are nearly closed in blank and NC-siRNA groups, while that in GRK2-siRNA group remained apparent distances. While lines, wound edges. Scale bar, 100 μM. **(B)** Migration ability tests on HTR8/SVneo cells with GSK180736A at different concentrations (0.5, 1, 2.5, and 5 μM) for 24 h. Scale bar, 100 μM. **(C)** Representative images of invaded HTR8/SVneo cells through chambers’ membrane (100×). The histograms show apparently reduced percentage of invasive cells in GRK2-siRNA group compared to others, which should be interpreted more than necroptosis. **(D)** Representative images and the analysis of EVTs migration ability tests performed on human villous explants. **(E)** Representative images of invaded through chambers’ membrane(x100). HTR8/SVneo cells treated with GSK180736A at different concentrations (0.5μM, 1μM, 2.5μM, 5μM) for 24 hrs and invaded through chambers’ membrane(100x). **(F)** HTR8/SVneo cells administrated with z-VAD-FMK (25μM, 24 hrs) or nec-1 (30 μM, 24 hrs) cannot restore the migration ability of cells. Note: NS, not significant; **P <0.01, ***P <0.001, ****P <0.0001.

Compared to the negative control (NC-siRNA) treatment, down-regulation of GRK2 markedly reduced the migration capacity (Figure 7A) and invasion ability (Figure 7C) of the HTR8/SVneo cells by 86.8 and 69.9%, respectively. Consistent with this, the migration ability (Figure 7B) and invasion capability (Figure 7E) reduced inversely correlated to the dose of GSK180736A. Notably, based on the previous findings that approximately 30% of cells were necrotic after 48 h post-transfection, we hypothesized that the knockdown or inhibition of GRK2 could attenuate the invasion and migration capability even if excluding excessive cell death, which may explain the poor spiral artery remodeling in GRK2-deficient mice. Villous explants were cultured and classified into 2 groups for testing the impact on EVTs’ migration, where the GSK180736A remarkably retained the expansion of EVTs (Figure 7D). Besides, we administrated caspase inhibitor z-VAD-FMK (z-VAD) or the necroptosis inhibitor necrostatin-1 (Nec-1) to *GRK2-*knockdown cells to see whether the impaired invasion capacity of the HTR8/SVneo cells could be restored by these inhibitors (Figure 7F). However, no significant difference in invasion ability was observed between groups with/without the treatment of inhibitors. Based on these, we reasoned the compromised spiral artery remodeling might be impaired by the death of invasive trophoblasts and the invasion incapacity mediated by GRK2 deficiency. Hence, these observations emphasized the crucial roles of GRK2 in the pathogenesis of preeclampsia by initiating trophoblast necroptosis and interfering with the trophoblastic invasion ability.

## Discussion

Preeclampsia is more than merely a “hypertensive disease” of pregnancy, as it is more commonly postulated to be a “trophoblastic disease” in its origins (Staff, 2019). Although this condition is recognized as a sequel to placenta malformation, the factors contributing to poor placentation remain unclear. GRK2 is known to play a critical role in controlling blood pressure. Herein, we have also found that GRK2 expression is involved in trophoblast survival and placenta development and that GRK2 levels are significantly downregulated in placentae in term PE. Then we developed murine models in mice with the placental absence of GRK2; we found that the placental labyrinth layers were severely damaged, and the spiral arteries were extremely small. In particular, focal regions of necrosis and reduced activation of caspase were identified. As necroptosis can trigger localized inflammatory responses and organ injury, we hypothesized that trophoblast necroptosis, not apoptosis, caused poor placentation and oxidative stress in preeclamptic placentae (Eppensteiner et al., 2018; Reis et al., 2020). In addition, the mice receiving 4 mg/kg GSK180736A daily from E8.5 to E13.5 and the mice intrauterine injected with GRK2-lentivirus at E8.5 developed PE-like phenotype: new-onset hypertension, proteinuria, and increased serum angiogenic cytokines from E14.5-E18.5. *In vitro*, when GRK2 is genetically ablated or pharmacologically inhibited by GSK180736A, the immortalized human extravillous cytotrophoblasts cell line, HTR-8/SVneo, was susceptible to RIPK1/RIPK3/MLKL-dependent necroptosis. Besides, inhibition of necroptosis by Nec-1 prevents the excessive trophoblast cell death in HTR8/SVneo cells, proven by the integrity of the nuclear envelope and the increased ratio of cell survival. Moreover, increased necrosome activity and disruption of trophoblast membranes were confirmed by co-immunoprecipitation (Co-IP), immunoblotting (IB) and electron microscopy. We further investigated the mechanisms behind insufficient spiral artery remodeling, and found the absence of GRK2 impaired the remodeling process by excessive death of trophoblast giant cells. Overall, this study has demonstrated a causal role of trophoblastic GRK2 deficiency in the onset and progress of PE by RIPK1/RIPK3/MLKL-dependent necroptosis and impaired invasion capability.

Prior work has established the potential role of GRK2 in cell death through mitochondria-dependent apoptosis. In ischemic cardiomyocytes, enhanced mitochondrial localization of GRK2 can induce pro-death signaling (Chen et al., 2013). In chronic heart failure, increased levels of GRK2 promote the desensitization and down-regulation of the β adrenergic receptor (βAR), induce cardiac insulin resistance, and reduce free fatty acid utilization, increase catecholamine secretion from the adrenal gland and aldosterone level through β-Arrestin (Ciccarelli et al., 2020). In acute ionizing radiation exposure-damaged mitochondria, GRK2 deletion accelerates the deteriorative processes of mitochondrial fission/fusion, initiating the extrinsic pathway of apoptosis (Franco et al., 2018). In lymphoma cells, knockout of GRK2 leads to enhanced MALT1-mediated IκB phosphorylation, RELB and CYLD cleavage, cytokine secretion, and cell proliferation, and GRK2 rescue reverses these effects. Thus, Cheng et al. (2020) suggested GRK2 as a lymphoma suppressor in inhibiting CARMA1-BCL10-MALT1 complex formation. These results elucidate GRK2 as a stress-sensor protein to determine cell fate following exposure to ischemia or harsh conditions, or as the upstream molecules in canonical NF-κB signaling. However, these studies haven’t identified role of GRK2 in modulating non-apoptotic cell death patterns, such as necroptosis, ferroptosis, pyroptosis, and entosis.

Non-apoptotic cell death signaling pathways have been shown to mediate placental ischemia and oxidative stress in PE placentae (Fisher, 2015; Cheng et al., 2020). Cheng et al. (2019) proposed placental pyroptosis as an inducer releasing angiogenic factors into the maternal circulation, potentially contributing to early-onset PE pathology. They reasoned the pathological stimuli such as hypoxia can disrupt placental ER homeostasis, leading to ER stress and the activation of unfolded protein response (UPR), which could activate the NOD-like receptor pyrin-containing 3 (NLRP3) inflammasome in trophoblasts. Another study found that inhibition of miR-30b-5p expression and supplementation with ferroptosis inhibitors attenuated oxidative stress in rat placentae; therefore, it was suggested that inhibition of miR-30b-5p could be a potential therapeutic target for PE by maintaining the expressions of 3′-UTR of Pax3 and SLC7A11 mRNA and preventing the depletion of GSH (Zhang T. et al., 2020). Previous *in vitro* findings have shown increased necrosome levels and necroptotic events, as visualized by TEM imaging in primary cytotrophoblasts from early-onset preeclamptic (E-PE) placentae (Bailey et al., 2017). They first proposed that necroptosis may contribute to both early-onset PE (E-PE) and late-onset PE (L-PE), while the necroptosis in E-PE is stimulated by oxidative stress like ceramide accumulation, whereas the necroptosis in L-PE results from cellular senescence and aging. They first reported the reduced caspase-8 activity in E-PE placentae may take trophoblasts more susceptible to necroptotic cell death in this pathological scenario.

In contrast, our results are based on L-PE patients and mice models. From the *in vitro* experiments, *ex vivo* villous explants and *in vivo* mice experiments, we demonstrate that (1) trophoblast cells, especially EVTs, express a considerable level of GRK2, (2) GRK2 deficiency is a powerful inducer of necroptosis in trophoblasts, (3) L-PE placentae are susceptible to necroptosis, not the caspase-dependent apoptosis signaling pathway. This GRK2 induced necroptotic mechanism can be prevented using Nec-1. (4) GRK2 deficiency induces para-vascular necroptosis, which then leads to fewer active TGCs at decidual portion and results in poor spiral artery remodeling in a dose-dependent pattern, (5) GRK2 deficiency in placenta is sufficient for the development of hypertension, renal dysfunction, and elevated angiogenic cytokines at late gestational age. The severity of clinical features positively correlates to the extent of GRK2 deficiency in a dose-dependent manner.

Using primary cells to undertake *in vitro* experiments was impracticable in this study, due to the renowned difficulty of obtaining pure, primary, first-trimester, human trophoblasts. Abbas et al. (2020) have conducted published research over the past 5 years, yet only 76/1044 studies used primary human EVTs. The HTR8/SVneo cell line was the first EVT cell line to be developed by Graham et al. from the first-trimester human placenta and remains the most commonly used cell line to study EVT invasion, proliferation, and regulation in the present day (Ding et al., 2016; Muschol-Steinmetz et al., 2016; Yang et al., 2016). Other choriocarcinoma-derived human placental cell lines are BeWo, JEG3, and JAR, but these are highly malignant, containing abnormal numbers of chromosomes and substantially different transcriptomic profiles than EVTs (Gierman et al., 2015; Abou-Kheir et al., 2017). In recent years, 3D organoid culture systems have emerged as a promising platform to study microenvironments of human organs (Neal et al., 2018). In 2018, the first trophoblast organoid culture system was established as a new model for maternal-fetal interaction investigations (Turco et al., 2018). These trophoblast organoid cultures have been used to study immune responses against human cytomegalovirus (HCMV) and Zika virus (ZIKV) at the maternal-fetal interface (Parker et al., 2020). Future studies should use 3D extracellular matrix to dissect the relationship between excessive death of EVTs and EVT failure to breach spiral arteries, in real-time.

In the current study, mice models received an intrauterine injection of lentivirus. In future investigations, we require alternative strategies to precisely examine the role of GRK2 deficiency in a trophoblast-specific manner. Adeno-associated virus (AAV) gene therapy is infeasible for the present study because AAV-2 induces placental dysfunction (Arechavaleta-Velasco et al., 2006). Besides, AAV-2 is highly cytotoxic to early-stage embryos, and could potentially result in spontaneous abortion (Gordon, 2002; Naiche and Papaioannou, 2007). Since constitutively high expression of CRE may cause fetal development defects (Tobita et al., 2017), the use of LV-Cre for placental conditional gene knockout is complicated. However, micro-injection of lentiviral vector to blastocysts could be an alternative method (Ueno et al., 2013). Zhang et al. (2015) have reported that the trophectoderm of Zona-free blastocysts could be infected with lentiviruses, which would bring about a paradigm shift in the tools used to study trophoblast invasion. These novel models could be considered in future investigations to expand our understanding of this field.

Finally, we propose a putative model of necroptosis in PE. Downregulation of GRK2 induces necroptosis, which then triggers trophoblastic debris, bioactive cytokines into circulation, and finally leads to hypertension and extensive targeted organ damage. Overall, by targeted modulating the levels of GRK2 or blocking the necroptotic signaling, we may develop alternative therapeutic avenues for PE in blocking excessive necrosis, suppressing deadly inflammatory pathways and preventing newborn deaths owing to prematurity preterm births.

## Data Availability Statement

The original contributions presented in the study are included in the article/Supplementary Material, further inquiries can be directed to the corresponding author/s.

## Ethics Statement

The studies involving human participants were reviewed and approved by Institutional Review Committee of Chongqing Medical University. The patients/participants provided their written informed consent to participate in this study. The animal study was reviewed and approved by the Ethics Committee of Animal Experiments of Chongqing Medical University (SYXK-YU 2018-0003). Written informed consent was obtained from the individual(s) for the publication of any potentially identifiable images or data included in this article.

## Author Contributions

H-BQ conceived and supervised the project. ZL and L-LX designed the research and performed cell experiments. ZL performed mice mating, blood pressure tests, and mice model establishments on ICR mice. ZL and XQ evaluated the mRNA level of GRKs on human placentae. XQ, HZ, and XL performed the kidney histology. ZL and L-LX constructed the manuscript with input from H-BQ, HZ, and XL. All authors contributed to the article and approved the submitted version.

## Conflict of Interest

The authors declare that the research was conducted in the absence of any commercial or financial relationships that could be construed as a potential conflict of interest.

## Publisher’s Note

All claims expressed in this article are solely those of the authors and do not necessarily represent those of their affiliated organizations, or those of the publisher, the editors and the reviewers. Any product that may be evaluated in this article, or claim that may be made by its manufacturer, is not guaranteed or endorsed by the publisher.
