# Supplementary material for: Role of GRK2 in Trophoblast Necroptosis and Spiral Artery Remodeling: Implications for Preeclampsia Pathogenesis
Source: Front Cell Dev Biol. 2021 Nov 30;9:694261. doi: 10.3389/fcell.2021.694261 (PMC8670385; doi:10.3389/fcell.2021.694261)

Supplementary Tables

Supplementary Table s1 | Clinical Characteristics of patients who provided placenta extracts

|  | **Normotensive (n=25)** | **Severe Preeclampsia (n=26)** |
| --- | --- | --- |
| Maternal Age (yrs) | 29.96±0.6595 | 28.81±0.6127 |
| Gestational Age (wks) | 37.52±0.1172 | 36.81±0.1841** |
| Systolic Blood Pressure (mmHg) | 112.8±2.299 | 165.1±2.009**** |
| Diastolic Blood Pressure (mmHg) | 71.52±1.698 | 88.65±2.747**** |
| Proteinuria (24hUP) | 0.09200±0.01143 | 3.615±0.1120**** |

Data are expressed as mean ± SEM. GA and proteinuria were analysed by Mann Whitney U-test; all other variables were analysed by student’s t-test. **P < 0.01, ****P < 0.0001, versus normotensive.

**Supplementary Table s2 | Clinical Characteristics of patients who provided villi extracts**

|  | **Normal (n=8)** |
| --- | --- |
| Maternal Age (yrs) | 24.56±1.119 |
| Gestational Age (wks) | 9.333±0.8660 |
| Systolic Blood Pressure (mmHg) | 106.9±2.131 |
| Diastolic Blood Pressure (mmHg) | 65.78±1.479 |

Data are expressed as mean ± SEM.

**Supplementary Table s3 | Forward and reverse oligonucleotide primer sequences used for analysis of expression of GRK1-GRK7, and Beta-actin in human placenta**

| **Primer Name** |  | **Sequence (5’ to 3’)** |
| --- | --- | --- |
| GRK1 human | forward | CTTTCAGCAGTTCCTACAATCG |
|  | reverse | TCCTTAAACTTCGCCACTATCC |
| GRK2 human | forward | ATGCATGGCTACATGTCCA |
|  | reverse | ATCTCCTCCATGGTCAGCAG |
| GRK3 human | forward | GCAGTGCCGACTGGTTCT |
|  | reverse | GTCTGAAAGGGCTGTGACCT |
| GRK4 human | forward | GGGACTGAAGGAGGAGAACC |
|  | reverse | TTTGTTACGGGTTGCCTTTC |
| GRK5 human | forward | CACCGGAAGCAGCTAGTTTC |
|  | reverse | TTTTTTGATAACCCCCTCCC |
| GRK6 human | forward | CCTCGAGCGTGACTATCACA |
|  | reverse | CGGTATTGCCTGAAGGTGTT |
| GRK7 human | forward | AATGGTTACATGGCTCCTGAGATCCT |
|  | reverse | AGGCCACGTTCCAGGCGAGGAAAGTT |
| Beta-actin human | forward | CATGTACGTTGCTATCCAGGC |
|  | reverse | CTCCTTAATGTCACGCACGAT |

**Supplementary Figures**

**FIGURE S1 | Expression of other GRKs subtypes in human placenta**

**(A-F)** the RT-PCR analysis of GRKs’ gene expression in patient placentas. The GRK2 mRNA analysis have shown in Fig 1A. **(G)** Western blotting analysis of the most common GRKs’ subtypes in patient placentas. Note: ns, not significant.

**
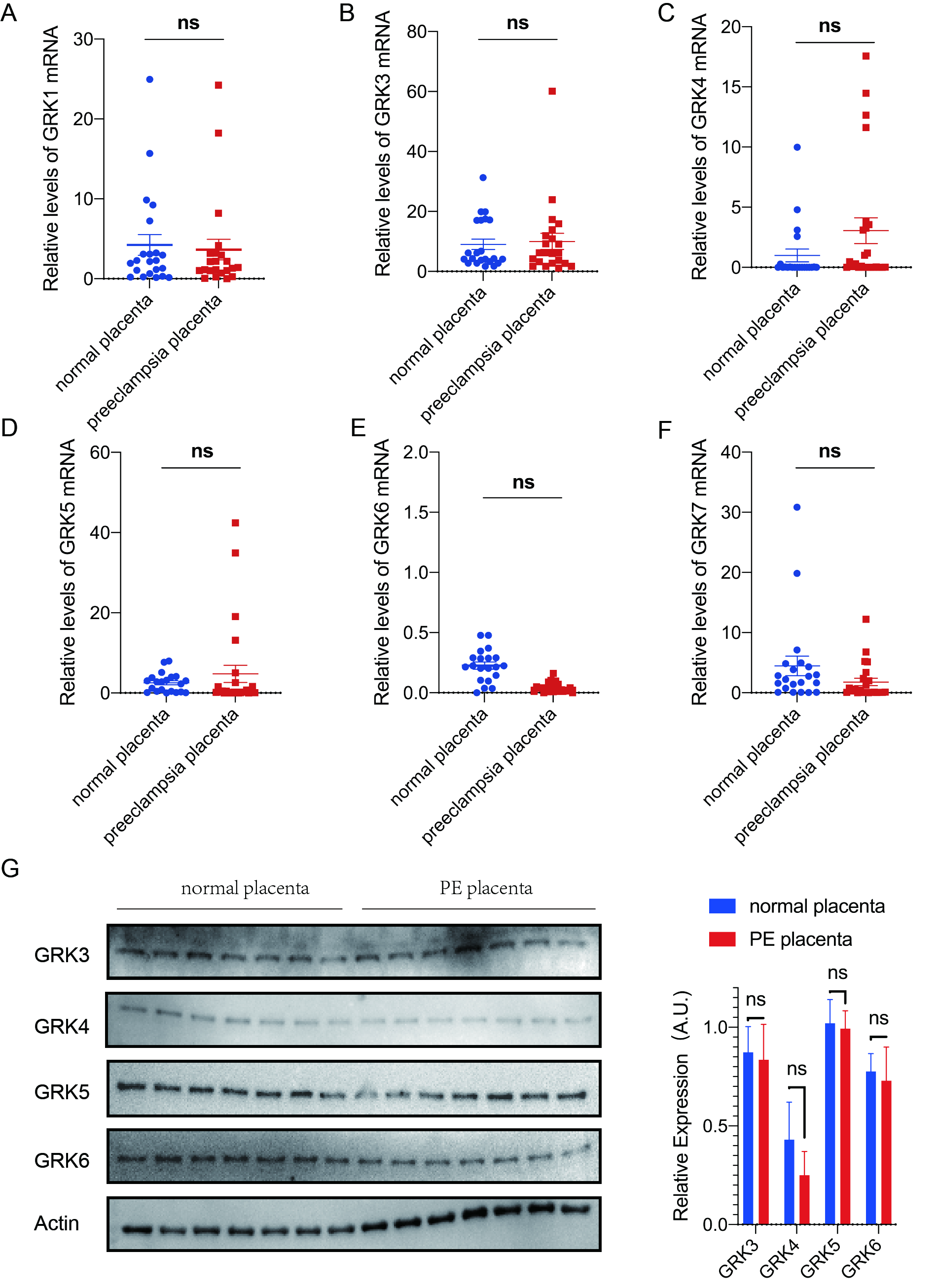
**

**FIGURE S2 | GRK2 is indispensable for placenta formation in mice and patients.** **(A)** Spatio-temporal pattern of the GRK2 contents in WT mice uterus assessed by immunofluorescence staining. Dec. indicates decidua; EPC, ectoplacental; Sp, spongiotrophoblast; Lab, labyrinth; YS, yolk sac. *Scale bar, 400μm.* **(B)** Representative images of immunofluorescence stained with GRK2(red) and CK7(green; trophoblasts) in human 1^st^ villi columns. Gestational weeks from 6 wks to 13wks. The magnification was 100× (upper panel, *Scale bar,100μm*) and 400× (lower panel, *Scale bar,20μm*). The lower panel is the magnification of the indicated area (white box) of the upper panel.

**
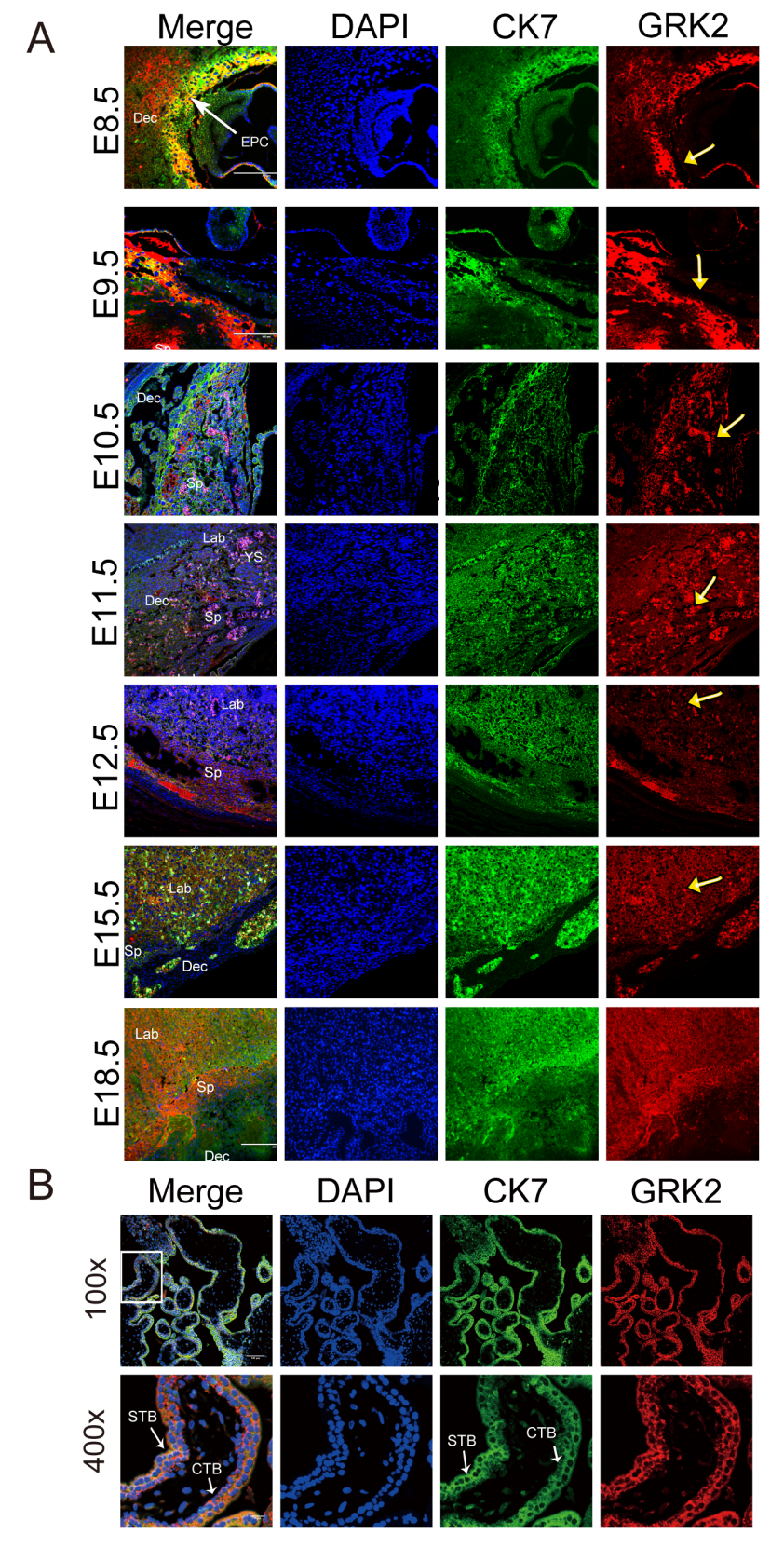
**

**FIGURE S3 | Blood pressure and urine protein of *GRK2*-KD shRNA treated non-pregnant mice. (A)** The blood pressure of mice who intrauterine injected with G*RK2*-shRNA. **(B)** The 24-hrs urinary proteins in lentivirus treated groups. **(C)** The glomerular histopathology was assessed by HE (left panel) and PAS (right panel) staining. All the lower left box shows the magnification (*Scale bar,10μm*) of the smallest square box in each large rectangle, *Scale bar*, *100μm*. No significant renal histology malformation is observed in non-pregnant mice 10 days after GRK2-KD shRNA treated. Note：The non-pregnant mice are set as control group. NS, not significant, ^###^ *P* < 0.001, ^####^ *P* < 0.0001 versus *GRK2*-KD shRNA treated pregnant group.


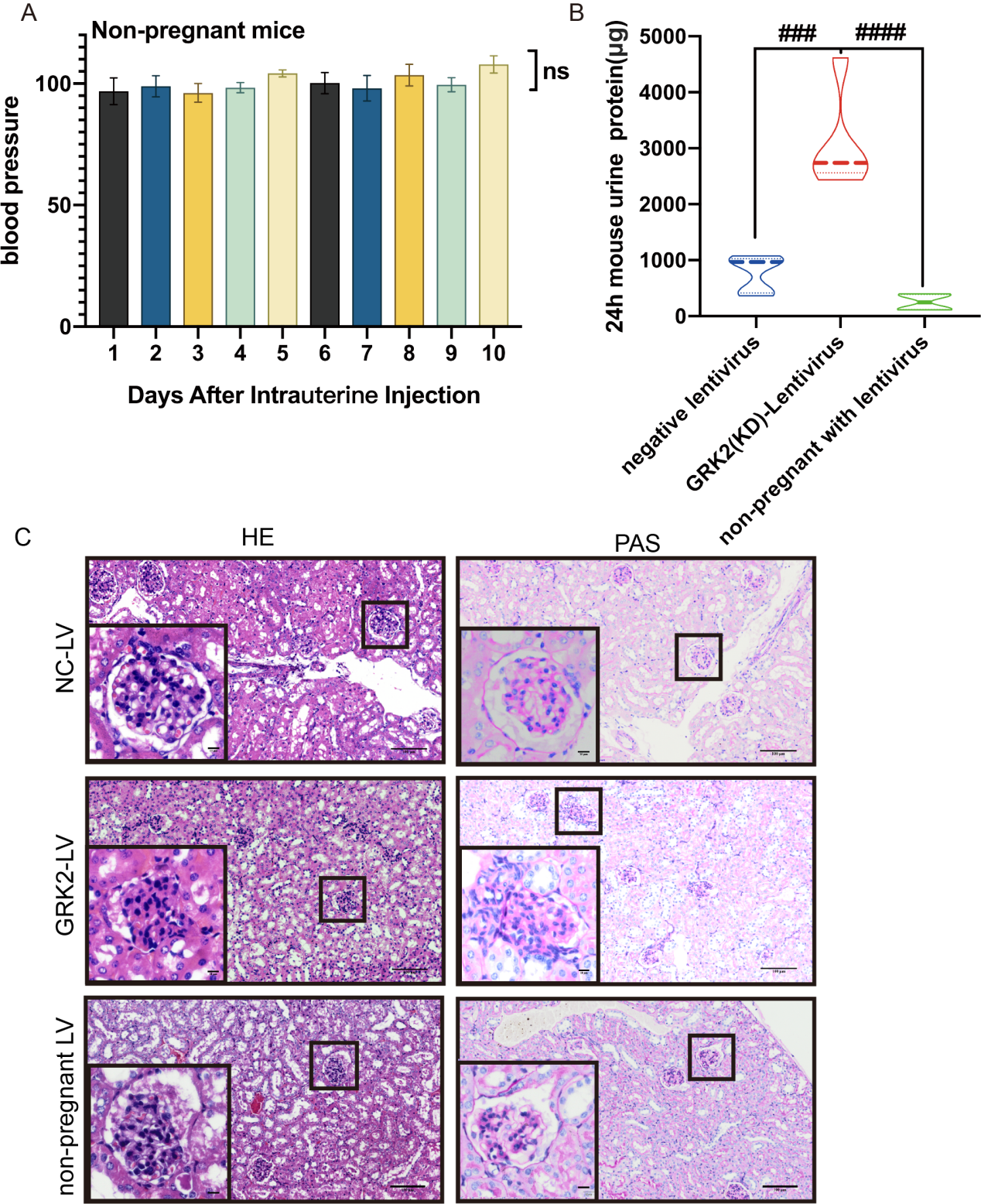


**FIGURE S4 | Mass proteinuria is a crucial clinical feature of *GRK2*-KD shRNA treated pregnant mice, which identical to PE patients. (A)** The 24hrs urine was collected at E16.5 by metabolism cage, which was frequently around 2-3 ml/24hrs in NC-LV treated mice, but 20-40ml/24hrs. **(B-C)** in GRK2-LV treated mice at E16.5. This validated the permeability diuresis in GRK2-LV treated mice, which can attract the urine and albumin loss. This is assembled to the crucial clinical feature of PE patients.


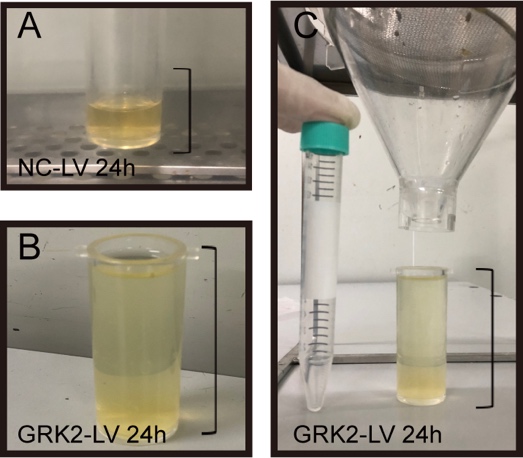


**FIGURE S5 | The transfection efficiency was validated in placentas before (E8.5) shRNA intrauterine injection and after (E11.5). (A-B)** The GFP staining indicating lentivirus expression, *Scale bar, 500μm*.


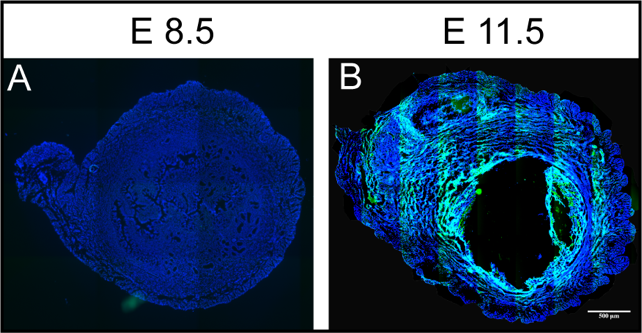


**FIGURE S6 | Suppression of GRK2 in placenta impairs spiral artery remodeling by inducing extensive trophoblasts’ death. (A)** 1^st^ panel: Co-staining of CD34 (green signals) and Plfr (red signals), and nuclei in E18.5 decidual-placental interface. Decidual vessel density and TGCs density are remarkably reduced in 4mg/kg group and GRK2-LV group, compared to other groups. 2^nd^ panel: Co-staining of GRK2 (green) and p-MLKL(S358) (red) and nuclei in E18.5 decidual-placental interface. The para-vascular tissue in GRK2-deficient groups are lack of green signals and encircled with necrotic signals. Arrows indicate micro-vessels at deciduae. *Scale bar, 50μm*. **(B)** Co-staining of GRK2 (green) and p-MLKL(S358) (red) and nuclei at the decidual para-vascular tissue from PE patients and controls. *Scale bar, 50μm*. **(C)** TEM images show typical necrotic cells surrounding the decidual arteries from PE patients. From left to right, 3000x, 6000x and 15000x magnification respectively. **(D)** Co-staining of CD68 (macrophage) and Ly6G (neutrophils), and nuclei at E18.5 embryos. Macrophage and neutrophil infiltration at GRK2-deficient deciduae are illustrated by arrows. *Scale bar, 50μm*.


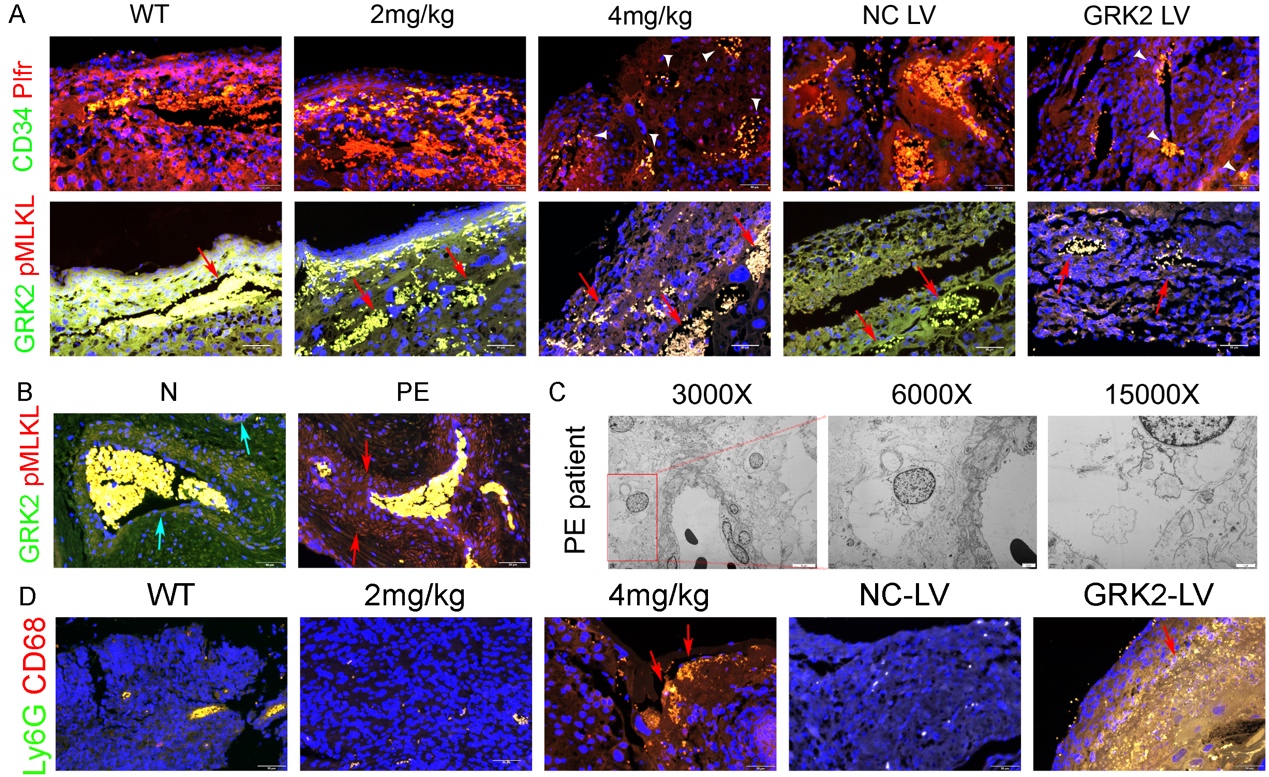

Supplement: Supplementary file 1 [file Table_1.DOCX]
